# Effects of working memory load on uncertain decision-making: evidence from the Iowa Gambling Task

**DOI:** 10.3389/fpsyg.2015.00162

**Published:** 2015-02-19

**Authors:** Ji-Fang Cui, Ya Wang, Hai-Song Shi, Lu-Lu Liu, Xing-Jie Chen, Ying-He Chen

**Affiliations:** ^1^National Institute of Education SciencesBeijing, China; ^2^Institute of Developmental Psychology and School of Psychology, Beijing Normal UniversityBeijing, China; ^3^Neuropsychology and Applied Cognitive Neuroscience Laboratory, Key Laboratory of Mental Health, Institute of Psychology, Chinese Academy of SciencesBeijing, China; ^4^North China Electric Power UniversityBeijing, China; ^5^University of Chinese Academy of SciencesBeijing, China

**Keywords:** Iowa Gambling Task, uncertain decision-making, working memory, somatic marker hypothesis, implicit learning

## Abstract

The Iowa Gambling Task (IGT) simulates uncertain gains and losses in real life situations and thus is a good measure of uncertain decision-making. The role of working memory (WM) in IGT performance still remains unclear. The present study aimed to examine the effect of WM on IGT performance. Three groups of participants matched on gender ratio were randomly assigned to no WM load, low WM load, and high WM load conditions. Initially the three groups did not show significant difference in WM capacity. They finished a modified version of IGT and then their implicit learning effect and explicit cognition on IGT were assessed. Results indicated a linear increasing trend of IGT performance among high WM load, low WM load and no WM load groups; participants in the no WM load and low WM load groups revealed implicit learning effect, while participants in the high WM load group did not; all participants showed explicit cognition on IGT to the same level. These results suggested that participants in the high WM load group showed good explicit cognition to IGT but showed poor performance. This pattern is similar to frontal patients. Further studies should be conducted to explore this issue.

## INTRODUCTION

### IGT AND UNCERTAIN DECISION-MAKING

Decision-making is closely related to people’s life, and usually a certain degree of uncertainty is involved in decision-making (Platt and Huettel, 2008). The Iowa Gambling Task (IGT) simulates the uncertainty of gains and losses in real life situations through the setting of monetary reward and punishments (Bechara et al., 1994). It is a good analogy of the uncertain decision-making in daily life and received much attention and studies since it is being developed. In the task, participants were presented with four decks of cards and asked to select cards from these decks to earn as much money as possible. Of these four decks, two show large immediate wins but sometimes even larger losses thus cause loss upon repeated plays in the long run, so they are disadvantageous decks. The other two decks show small immediate wins and also sometimes smaller losses thus cause win upon repeated choice in the long run, so these are advantageous decks.

Healthy participants developed anticipatory skin conductance responses (SCRs) when playing with bad decks before they had explicit knowledge of the advantageousness of the decks and could decide advantageously (Bechara et al., 1997). Bechara et al. (1994, 1997, 2000), Damasio (1994), Bechara and Damasio (2005) used the somatic marker hypotheses (SMHs) to explain the participants’ performance in IGT and they paid special attention to the guidance of somatic marker (emotional) signal which reflected the body state changes to decision-making behavior. Specifically, the bodily generated somatic signals such as SCRs were different to advantageous and disadvantageous decks before participants got explicit knowledge to the task, thus participants could make advantageous decisions. However, theoretical explanations to IGT are still under debate (see Dunn et al., 2006). Researchers represented by Maia and McClelland (2004, 2005) emphasized the role of cognitive processes in decision-making, such as working memory (WM). Previous studies showed inconsistent results on the role of WM in IGT performance (e.g., Hinson et al., 2002; Turnbull et al., 2005; Pecchinenda et al., 2006).

### WM IN IGT

The most direct evidence for the relationship between IGT and WM comes from dual-task paradigm studies in which the effect of WM load on IGT performance were explored. The earliest study was conducted by Hinson et al. (2002). They adopted a task similar to IGT but more difficult. The task included three decks of cards, one advantageous deck, one neutral deck and one disadvantageous deck. They set three WM load conditions: high WM load (keep five randomized digits in memory), low WM load (random number generation), and no WM load. Results showed that the participants performed poorer in the WM conditions, and the anticipatory SCRs were also impaired. The authors suggested that WM was helpful for the development of SCRs. With WM load, the somatic markers may not develop, thus the decision-making performance was impaired. In their following study (Jameson et al., 2004), they explored whether the secondary task affect IGT performance through the interference to the central executive or the phonological loop. They also used an adapted IGT (four decks of cards: one advantageous, one neutral and two disadvantageous decks). They set three interference conditions: no WM interference (press the digit key presented on the screen), phonological loop interference (pronounce “the” repeatedly), and central executive interference (to maintain random digits). Results showed that compared to central executive interference condition, participants made more advantageous choices in the other two conditions, and the performance increased with time. Anticipatory SCRs showed difference between “advantageous” and “neutral/disadvantageous” choices in the phonological loop interference and no WM interference conditions, but no such differences were found in the central executive interference condition. The authors concluded that the central executive resources were required for the development of somatic makers. If the central executive is interrupted, somatic markers would not develop and IGT performance would be impaired.

In the studies of Hinson et al. (2002) and Jameson et al. (2004), they adapted the IGT to a large extent (for example, they only had 80 trials, they did not have two advantageous and two disadvantageous decks), and they used within subject design. Turnbull et al. (2005) used the standard IGT and set three conditions: no secondary task, phonological inhibition secondary task (report digits 1–9 in order), and central executive secondary task (random number generation). They used a between subject design, and results showed that these three conditions did not show significant differences in the rate of learning in the IGT. This result became the important evidence that the IGT performance was independent of WM interference. However, the no secondary task group showed a trend of better performance than the other two groups, which affected the power of their explanation.

For the reason of the inconsistent results, Pecchinenda et al. (2006) suggested that in previous studies the positions of decks were fixed, which may allow participants to use different strategies, such as maintaining the information of spatial positions of decks which would rely on WM function. In their study, they changed the positions of decks for each trial. Other parameters were the same as the standard IGT. They set two conditions: the high WM load condition in which they used the same task as in Hinson et al. (2002), i.e., to maintain random digits, and the low WM load condition in which participants needed to recall successive digits. This study used a between subject design. Results showed that in general the participants showed a linear learning trend, but only participants in the low WM load group chose advantageous decks more often. Their net score were significantly different from zero from block three, while participants in the high WM load group did not. The authors suggested that IGT performance depended on WM functions.

Other approaches were also used to examine the relationship between WM and IGT performance, such as relational studies. Toplak et al. (2010) reviewed studies that examined the cognitive explanations on IGT. Fifteen studies measuring both WM and IGT were reviewed, and only one study reported significant relationship between WM and IGT performance. Thus Toplak et al. (2010) suggested IGT performance was relatively independent of WM ability. Recently, Bagneux et al. (2013) adopted an individual difference approach to identify individuals with high and low WM capacity and asked them to conduct the IGT. The results showed that the high WM capacity individuals performed more advantageously in the IGT, which suggested the role of WM in uncertain decision-making.

Generally speaking, results on the role of WM in IGT performance were mixed. Damasio (1994), who proposed the SMH once suggested that one of the functions of somatic markers was to lead which choices need to be processed by WM, and allocate attention resources to them. In other words, successful IGT solution involves two steps. One was the generation of somatic markers to tell good or bad choice, and the other was to use these signals to allocate WM and attention resources to good choice. In this sense, the emotional and cognitive factors interacted in the decision-making process. From the neural basis perspective, the amygdale and ventromedial prefrontal cortex are reported to play an important role in the development of somatic makers. Brain lesion studies and neuroimaging studies in healthy participants showed that ventromedial prefrontal cortex, amygdale, and dorsolateral prefrontal cortex were involved in IGT performance (Bechara et al., 1994, 1996; Fellows and Farah, 2005a; Martinez-Selva et al., 2006). Studies also suggested that dorsolateral and ventrolateral prefrontal cortex were involved in WM performance (Wager and Smith, 2003; Owen et al., 2005). Therefore, the IGT and WM shared some neural mechanisms. Thus, if we add a WM load to the IGT, the performance of IGT might be affected.

### THE PRESENT STUDY

There are several limitations in the previous studies: first, participants were free to choose cards from any deck in the previous studies, thus participants might play perseveratively from the same deck instead of sampling more broadly. Past studies did not rule out the possibility that differences in IGT performance caused by WM load might be at least in part caused by WM load related search strategies rather than their decision-making abilities. Second, few studies have examined the effect of WM load on IGT performance, implicit learning, and explicit cognition in a single study, which makes the specific role of WM in IGT performance remains unclear yet. For example, whether WM load interferes the implicit learning, explicit cognition or other factors that affect IGT performance was not known.

The present study further investigated the effect of WM load on decision-making by using a dual task paradigm. The aim was to understand the role of cognitive resources (WM) on IGT performances. In order to overcome the influences of the searching strategies in decision-making process and to exclude the influence of participants’ willingness to explore all the decks (Cauffman et al., 2010), we used a single choice version of IGT (Cui et al., 2013, in preparation) in this study. In this version of IGT, participants were assigned a deck by the computer on each trial and required to make a decision whether to play with or pass that deck. The four decks were assigned to participants equally often. Therefore, the influence of WM load on searching strategies would be avoided. We also examined the effect of WM load on the implicit learning and explicit cognition of the IGT by including a second stage task and some questions. In the second stage, there was a free choice block without feedback for each trial but a total feedback for that whole block. This stage assessed participants’ implicit learning effect (Stocco and Fum, 2008). After finishing the second stage, participants were required to answer some paper-and-pencil questions on explicit knowledge of the IGT (Maia and McClelland, 2004; Guillaume et al., 2009).

Further adaptations were made on the task: first, it was argued that the reward and punishment schedule in the original IGT was too simple (Gozzi et al., 2011). Participants only needed to attend to the value of loss in the task, since the magnitude of wins were fixed (50 or 100) in the task (Cauffman et al., 2010). The present study made some changes on the magnitude of reward of each card, rather than adopting the fixed reward 50 or 100. Second, in order to avoid difficulties in explanation to a mixed reward and punishment in feedback, we adopted a net score feedback for each card (the feedback is either win or loss in each trial instead of presenting both win and loss to participants as in the original task). Third, Bagneux et al. (2013) suggested that increasing the total number of trials might allow participants to develop a tendency to make advantageous choices, so we included 200 trials in the first stage.

We held the following hypothesis: based on previous dual-task paradigm studies and theoretical analysis of SMH, we predicted that WM load would affect the learning process, and cause poorer IGT performance. Performance in the high, low and no WM load groups would show a linear increasing trend. We further expected that participants in the three conditions would show different implicit learning effect and explicit cognition on the IGT. Implicit learning and explicit cognition would also show a linear increasing trend in the high, low and no WM load groups.

## MATERIALS AND METHODS

### PARTICIPANTS

Ninety volunteers (45 males and 45 females) were recruited from university students in Beijing. They were aged between 18 and 27 (mean age 22) years old. All participants had normal or corrected to normal vision and free of neurological or psychiatric diseases. Participants were randomly assigned to three WM load groups (no WM load, low WM load, and high WM load) of IGT with equal gender ratio as van den Bos et al. (2013) suggested that there were gender differences in IGT performance. The three groups did not show significant difference in years of education [*F*(2,87) = 1.20, *p* = 0.305].

This study was approved by the Ethics Committee of Beijing Normal University. Participants were paid for their participation. In order to enhance the participants’ motivation, they were informed before the experiment that they would get additional reward according to their IGT performance. They had a minimal pay of 50 RMB, if their final earning was larger than 1000, then they would earn additional 5 RMB for every 500 points.

### EXPERIMENTAL TASKS

#### Iowa Gambling Task

**FIGURE 1:**
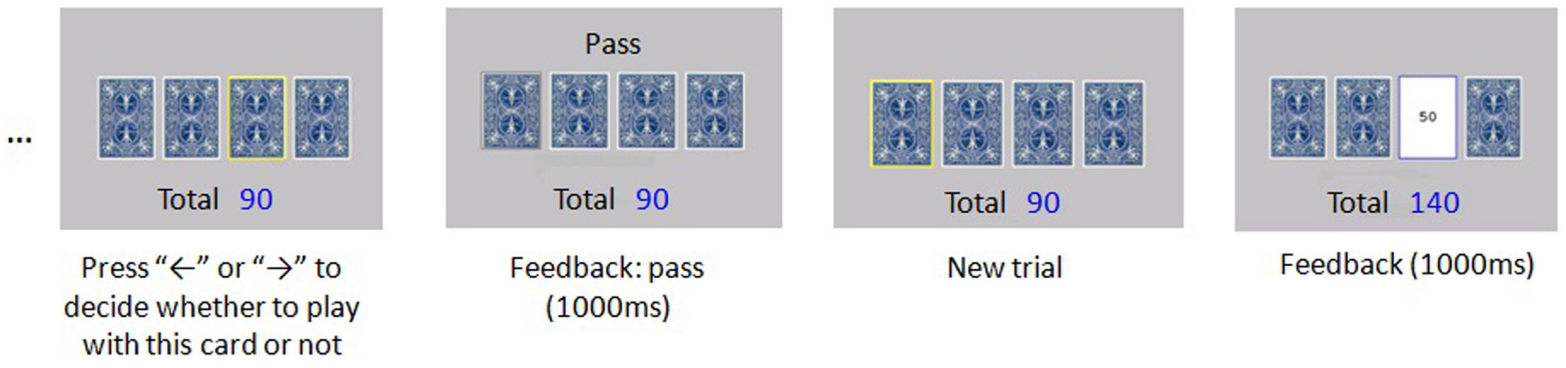
**Illustration of experiment flow**.

The original IGT was adapted in this study. The modified IGT consisted of two stages. The first stage took a single choice mode with feedback for each trial. In each trial, four decks of cards were presented on the screen, with one of them embedded in a yellow border, which indicated that one card from this deck was assigned by the computer. The participants were asked to decide whether to play with the card or not. If they decided to play, they should press the “←” key; if pass, press “→”. If the participants responded to play, the amount of reward or punishment for this trial (feedback) would be presented on the screen and added to the running total which was on the screen all the time. If the participants decided to pass, the feedback on the screen was “Pass”, with the running total unchanged. The feedback would be presented for 1000 ms. Then the next trial appeared. In each block (20 trials), five cards would be chosen from each of four decks. The four decks were assigned to participants at a random order. The task flow was shown in **Figure 1**.

Participants were made clear before the task that each deck had its own reward and punishment rules, and contained enough cards. Each time the computer would randomly select a card from the designated deck. If the participants chose to play with a card, that card would be turned over and taken out of that deck; if the participants chose to pass a card, that card would not be turned over and none would be taken away. Since there were plenty of cards in each deck, the number of plays would not affect the reward and punishment rules of a given deck. The payoff schedule was a bit different from the original IGT and was shown in **Table 1**. Specifically, the reward was not fixed to 100 or 50 but varied. Instead of presenting reward and punishment at the same time, we only presented the net reward or punishment to participants. The card was set at a fixed pseudorandom order. The card passed by participants still remained on the top of that deck. If the participant was assigned the same deck the next time and chose to play, it was still that card. This setting was to ensure that the expected value of each deck was consistent with the original design, and kept the same for all participants. However, the participants were not aware of this design. The total number of trials was not told to participants either.

**Table 1:** Payoff schedule in the adapted IGT.

Payoff schedule	Deck			
	A	B	C	D
Range of gains	80∼130	80∼130	40∼70	40∼70
Range of losses	–50∼–250	–1150	–5∼–25	–200
Percentage of net gain	0.5	0.9	0.5	0.9
Percentage of net loss	0.5	0.1	0.5	0.1
Position of the first loss	3	9	3	10
Expected value of 10 consecutive choice	–250	–250	200	250

*The outcomes of Deck A include: 100, 100, 80, 90, 130, –50, –100, –150, –200, –250. The outcomes of Deck B include: 100, 100, 100, 100, 80, 80, 90, 120, 130, –1150. The outcomes of Deck C include: 50, 50, 40, 60, 70, –5, –10, –15, –15, –25. The outcomes of Deck D include: 50, 50, 50, 50, 40, 40, 40, 60, 70, –200.*

In order to get enough number of plays by participants, the first stage included 200 trials according to the ratio of play in Cauffman et al.’s (2010) study. Then the second stage began. This stage was to assess the implicit learning effect. Based on Stocco and Fum’s (2008) study, this stage included a free choice block. In this stage, the computer did not assign a deck for participants to play or pass. Instead, the participants were asked to freely choose cards from any deck for 20 times. And they did not get feedback for each selection but only a total feedback for these selections at the end of this stage.

After the IGT, participants were required to fill a paper-and-pencil questionnaire, this was to assess their explicit cognition on IGT. Based on Guillaume et al.’s (2009) study, it included questions from four aspects: (1) What do you know about this task? (2) Do you find differences among these decks? (3) Suppose you will pick ten new cards from Deck A/B/C/D, will you win or lose? (one question for each deck) (4) If you were given another chance to play this game, but you can only choose from one deck, which deck will you choose from in order to win as much as possible? For questions (1) and (2), correct judgments of advantageousness of each deck scored 0.5; correct descriptions of win or loss of each deck scored 0.5; for question (3), each correct answer scored 1; for question (4), if the participants chose advantageous deck (C or D), they would get 1 score; if they chose disadvantageous deck (A or B), they would not get score. As a result, the maximum score of explicit cognition on IGT was 9.

WM load was manipulated through a dual-task paradigm by embedding a secondary digit recall task to the IGT. There were three WM load conditions: no WM load, low WM load and high WM load conditions. The no WM load condition was described as above. In the low WM load condition, the participant needed to keep 3 random digits in memory, such as “275”, while in the high WM load condition, the participant needed to keep 7 random digits in memory, such as “8129365”. When the participant was prompted to recall the digits, he/she needed to input the digits in order through keyboard. A string of digits was presented on the computer screen before the decision on cards started. When the participant remembered the digit string, he/she could press any key to start the first stage of the IGT. Then he/she needed to recall the digit string after 10 trials of card selection. After he/she recalled the digit string they remembered last time, a new digit string was presented on the screen for him/her to remember. Participants needed to recall the digit strings every 10 trials of card selection. In the WM load groups, both stage one and stage two contained this secondary task. Before the formal experiment, there was a practice on the secondary task (digit string recall task). The accuracy of the secondary task is the proportion of correctly recalled digits (with correct position) to the total number of digits needs to be memorized.

#### WM task used to compare participants’ WM capacity in different groups

The calculation span task modified from Grant and Dagenbach (2000) was used in the present study to assess participants’ WM capacity. The main purpose of including this task was to confirm the participants that randomly assigned to different conditions did not show significant difference in WM capacity. There was addition or subtraction formula presented at the center of the computer screen. The numbers in the formula were no larger than 15, and the participants were required to determine whether the formula was correct or not (press “←” if correct and press “→” if incorrect). At the same time, they should remember the second number in the formula. Each formula presented no longer than 4000 ms. Once the participant made a response, the next formula appeared. After a consecutive set of formulas presented, the participant was required to recall the numbers he/she just remembered in order by inputting them with keyboard. Formulas were generated by computer randomly with the following rules: the second number was different from the result number; and the second numbers in two consecutive formulas were different.

The number of formulas in each set ranged from 2 to 7. Each contained three sets, resulted in a total of 18 sets. The participants got one score if they correctly recalled one number (with correct position). The score ranged from 0 to 81. Two sets of practice were conducted before the formal test.

### PROCEDURE

After a general introduction to the experiment, the participants were free to ask questions. The formal experiment began after participants signing the informed consent form. The participants were tested individually. They finished the IGT first. After a rest, they were administered the WM task. The order of the tasks was the same as in Bagneux et al. (2013).

### DATA ANALYSIS

First, WM task performance was compared among the three groups to examine whether they showed significant difference in WM capacity. Second, as it was usual for IGT, participants’ performance was measured by dividing the trials into blocks of 20 consecutive choices, and calculating the block net score (subtracting number of plays for disadvantageous decks A and B from number of plays for advantageous decks C and D). The block net score was compared among different WM load groups. Third, the choices on each deck among different groups were compared. Fourth, the implicit learning effect in stage two was compared among groups. Fifth, the explicit cognition of IGT was compared among groups. In these analyses, for the block effects and interaction effects, Greenhouse–Geisser correction was used to adjust degrees of freedom. Based on results in the previous studies, we expected a linear trend in IGT performance among the high WM load, low WM load and no WM load groups, thus we made a planned linear contrast after examining the main effect of WM load.

## RESULTS

### COMPARISON OF WM PERFORMANCE FOR PARTICIPANTS IN DIFFERENT WM LOAD GROUPS

This study aimed to explore the effect of WM load on IGT performance. We compared the participants’ WM capacity to exclude its confounding on IGT performance. The scores of WM task (calculation span) were *M* = 77.9 (SD = 5.45) for the no WM load group; *M* = 78.8 (SD = 3.05) for the low WM load group;* M* = 78.0 (SD = 3.18) for the high WM load group respectively. One-way analysis of variance (ANOVA) showed that the difference was not significant, *F*(2,87) = 0.44, *p* = 0.645, $\eta_{p}^{2}$ = 0.010.

### COMPARISON OF IGT BLOCK NET SCORE IN DIFFERENT GROUPS

To ensure participants in different conditions accomplished tasks according to task requirements, we compared their secondary task performance in the high and low WM load groups. Results showed that participants were highly accurate in recalling the digits, and the accuracy were *M*_low_ = 0.983 (SD = 0.020) and *M*_high_ = 0.975 (SD = 0.035) for the low and high WM load groups respectively. One-way ANOVA showed that the difference was not significant, *F*(1,58) = 1.36, *p* = 0.249, $\eta_{p}^{2}$ = 0.023. The result suggested that the manipulation of secondary WM task was effective.

**FIGURE 2:**
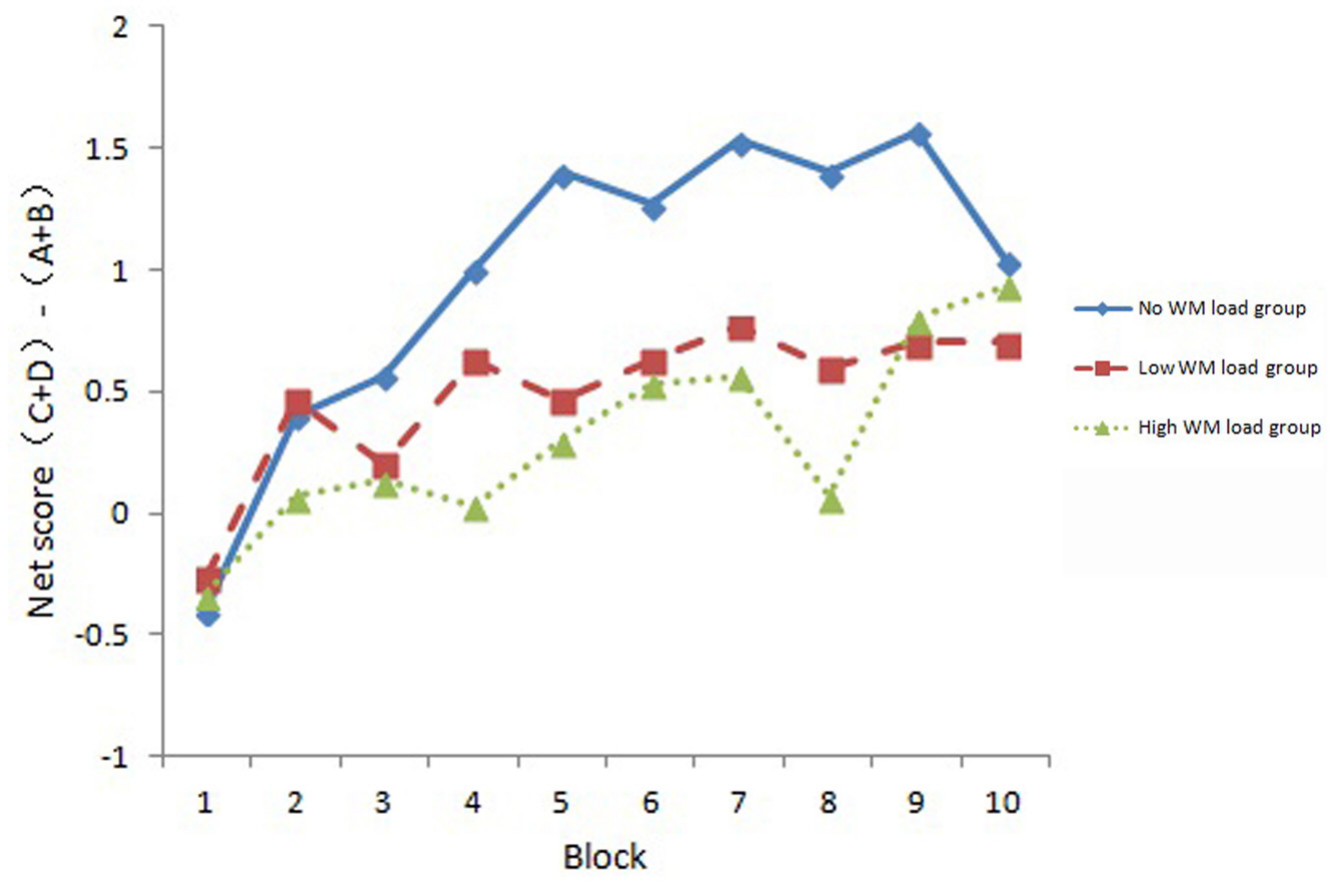
**Block net score in each WM load group**.

The block net score in each WM load group is shown in **Figure 2**. A 3 (WM load) × 10 (Block) mixed ANOVA was conducted with block net score as the dependent variable. Results showed that there was a significant main effect of Block, *F*(6.61,575.04) = 4.57, *p* < 0.001, $\eta_{p}^{2}$ = 0.050. There was a marginally significant effect of WM load, *F*(2,87) = 2.95, *p* = 0.057, $\eta_{p}^{2}$ = 0.064. Planned contrast showed a linear trend among the three conditions (the linear contrast was significant, *p* = 0.021, and the quadratic contrast was not significant, *p* = 0.534). The WM load × Block interaction was not significant, *F*(13.22,575.04) = 0.64, *p* = 0.871, $\eta_{p}^{2}$ = 0.014.

To further explore the learning effect in each group, we compared the net score of each block with 0. The results are shown in **Table 2**. Bonferroni correction was used for this analysis, thus the threshold for significance was 0.005. It can be seen that the no WM load group showed block net score larger than 0 for block 4, 5, 8, and 9. However, none of the low WM load or high WM load group showed a block net score larger than 0 after Bonferroni corrections. These results indicated that WM load influenced participants’ learning process of decision-making.

**Table 2:** Test of difference from zero for block net score in each WM load group.

Block	No WM load group			L ow WM load group			H igh WM load group		
	Net score *M*	*t*	*p*	Net score* M*	*t*	*p*	Net score* M*	*t*	*p*
1	–0.40	–1.29	0.206	–0.27	–0.59	0.558	–0.33	–1.06	0.300
2	0.40	0.97	0.339	0.47	1.08	0.291	0.07	0.23	0.821
3	0.57	1.95	0.061	0.20	0.71	0.483	0.13	0.54	0.595
4	1.00	**3.26**	0.003	0.63	2.00	0.055	0.03	0.10	0.921
5	1.40	**3.27**	0.003	0.47	1.85	0.075	0.30	1.03	0.313
6	1.27	2.57	0.016	0.63	2.35	0.026	0.53	1.39	0.174
7	1.53	2.62	0.014	0.77	2.09	0.046	0.57	2.66	0.012
8	1.40	**3.25**	0.003	0.60	2.07	0.048	0.07	0.27	0.787
9	1.57	**3.11**	0.004	0.70	2.46	0.020	0.80	2.05	0.050
10	1.03	1.63	0.114	0.70	1.77	0.087	0.93	2.94	0.06

*The significance of bold values is *p* < 0.005.*

### THE ANALYSIS OF CHOICE ON EACH DECK IN DIFFERENT GROUPS

**FIGURE 3:**
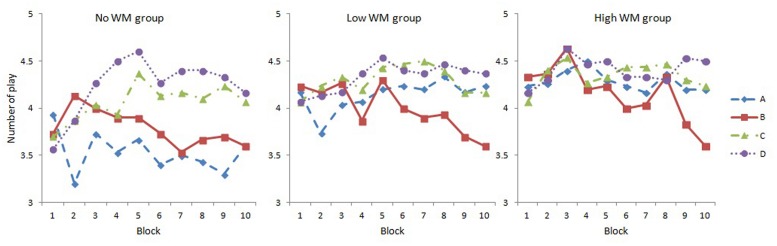
**Number of plays for each deck in each block under three WM load groups**.

A 3 (WM load) × 10 (Block) × 4 (Deck) mixed ANOVA was conducted, and results indicated that: the main effect of Deck was significant, *F*(3,261) = 14.55, *p* < 0.001, $\eta_{p}^{2}$ = 0.143. Further analysis revealed that number of plays in Decks C and D was significantly larger than that of Decks A and B (Bonferroni corrected). The main effect of WM load was significant, *F*(2,87) = 5.22, *p* = 0.007, $\eta_{p}^{2}$ = 0.107. Planned contrast indicated a linear trend among the three groups (the linear contras was significant, *p* = 0.002, and the quadratic contrast was not significant, *p* = 0.426). The Deck × WM load interaction was significant, *F*(4.70,204.34) = 3.11, *p* = 0.012, $\eta_{p}^{2}$ = 0.067; and the Deck × Block interaction was significant, *F*(17.42,1515.17) = 3.32, *p* < 0.001, $\eta_{p}^{2}$ = 0.037. **Figure 3** indicated that the trends of four decks changing over time were different among groups. The Deck × WM load × Block three-way interaction was not significant, *F*(34.83,1515.17) = 0.72, *p* = 0.887, $\eta_{p}^{2}$ = 0.016.

The simple effect analysis of Deck × WM load interaction revealed that the number of plays in Deck A was significantly different among WM load groups, *F*(2,87) = 9.63, *p* < 0.001. Planned contrast indicated a linear trend among the three groups (the linear contrast was significant, *p* < 0.001, and the quadratic contrast was not significant, *p* = 0.812). Decks B, C, and D did not show significant difference among groups.

The simple effect analysis of Deck × Block interaction revealed that the main effect of Block was significant in Deck B, *F*(6.19,550.68) = 4.40, *p* < 0.001, $\eta_{p}^{2}$ = 0.047, that participants tend to play less with Deck B over the course of the task. The main effect of Block was significant in Deck C, *F*(6.92,615.48) = 2.34, *p* = 0.024, $\eta_{p}^{2}$ = 0.026. Participants tend to play more with Deck C over the course of the task. The main effect of Block was significant in Deck D, *F*(7.21,641.87) = 4.87, *p* < 0.001, $\eta_{p}^{2}$ = 0.052, that participants tend to play more with Deck D over the course of the task.

From **Figure 3** and the above results we could find that the number of plays in Deck B decreased across blocks in each WM load group. At the same time participants showed a preference for Deck D, but for another disadvantageous Deck (Deck A), they were less played with only in the no WM load group.

### THE INFLUENCE OF WM LOAD ON IMPLICIT LEARNING EFFECTS

We analyzed participants’ choices in stage two in each WM load group. A one-way ANOVA was conducted to examine the effect of WM load on the net score of this block, and result revealed no main effect of WM load, *F*(2,87) = 0.82, *p* = 0.443. However, the comparison between net score and 0 indicated that the net scores in no WM load [*t*(29) = 2.65, *p* = 0.013] and low WM load [*t*(29) = 3.69, *p* < 0.001] groups were significantly different from 0, while in high WM load group, it was not significant different from 0 [*t*(29) = 1.28, *p* = 0.212].

A 3 (WM load) × 4 (Deck) repeated measures ANOVA revealed that the WM load × Deck interaction was not significant, *F*(5.51,239.51) = 1.50, *p* = 0.178, $\eta_{p}^{2}$ = 0.033, which meant participants showed relatively consistent trend in selecting cards among three WM load groups.

### COMPARISON OF EXPLICIT COGNITION ON IGT AMONG DIFFERENT GROUPS

The scores of explicit cognition on the IGT under different WM load were: *M* = 4.5 (SD = 2.50) for the no WM load group; *M* = 4.2 (SD = 1.63) for the low WM load group; *M* = 4.9 (SD = 2.17) for the high WM load group. One-way ANOVA showed that the main effect of WM load was not significant, *F*(2,87) = 0.82, *p* = 0.446, $\eta_{p}^{2}$ = 0.018. At the same time, the relationships between explicit cognition score and total net score in stage one was significant only in the no WM load group: *r* = 0.58, *p* < 0.001. For the low WM load group, *r* = 0.27, *p* = 0.156; for the high WM load group, *r* = 0.30, *p* = 0.111.

We also analyzed participants’ favorite deck (**Table 3**). Chi-square test revealed that the deck preference under different WM load groups was not significantly different, χ^2^ = 1.65, *p* = 0.949.

**Table 3:** Frequency of favorite decks reported by participants in each WM load group.

Group	Favorite deck				Sum
	A	B	C	D	
No WM load group	6	5	7	12	30
Low WM load group	7	5	6	12	30
High WM load group	5	3	8	14	30
Sum	18	13	21	38	90

## DISCUSSION

In this study we employed a modified version of IGT which could avoid the influence of WM load on searching strategies, and explored the effect of WM load on uncertain decision-making. The main findings were: the WM load had a marginal effect on IGT net score; further analysis indicated the IGT performance showed a linear increasing trend among high WM load, low WM load and no WM load groups. Participants in the no WM load group played less with Deck A than the low and high WM load groups; participants in the no WM load group played less with Deck B than the high WM load group. Participants in the three groups did not show significant difference on implicit learning effect. However, the no and low WM load groups showed a learning effect significantly different from 0, yet the high WM load group did not. All three groups reached a same level of explicit cognition on IGT. We discussed these results in turn.

### THE INFLUENCE OF WM LOAD ON IGT PERFORMANCE

The present study adopted a dual task paradigm to investigate the effect of WM load on IGT performance. The basic logic of dual task paradigm is that if the two tasks depend on the same cognitive resources, the secondary tasks would reduce cognitive resources available for the primary task, and as a result the performance of the primary task will be affected. In the present study, we employed a modified version of IGT which could avoid the influence of WM load on searching strategies which might be a confounding factor in previous studies. Analysis on block net score revealed that the main effect of WM load was marginally significant. Planned contrast revealed block net score showed a linear trend among the three groups which indicated an interference role of WM load on IGT performance. The interaction between Block and WM load was not significant. These results suggested that all the three groups showed a learning effect, which can be seen from **Figure 2**. We compared block net score in different WM load groups with 0, and results showed that block net score of the no WM load group was significantly higher than 0 from block 4 (except for block 6, 7, 10), while none of the block net score in the low WM load group or the high WM load group did so. This might mean the influence of WM load on the learning process of decision-making. It was consistent with previous studies that WM played a role in IGT performance (Hinson et al., 2002; Jameson et al., 2004; Pecchinenda et al., 2006; Bagneux et al., 2013). Nevertheless, these results were not consistent with some studies (Turnbull et al., 2005; Gozzi et al., 2011). This might be because we used different versions of IGT, and different secondary task were used. We used a single choice version of IGT that excluded the potential confounding of attention focus shifting and searching strategies (Cauffman et al., 2010), while previous studies used other versions of IGT. For the secondary task, the random number generation task used in previous studies can be self-paced and might be slowed down when WM was required, thus it might not be a good task taxing WM resources (Pecchinenda et al., 2006). But in the present study, participants needed to maintain a digit string all through the task and was a valid secondary WM task. Moreover, analysis of secondary task accuracy also revealed that it was high in both WM load groups (higher than 0.97). This indicated that participants performed the secondary task carefully according to task requirements, and it also suggested that the dual-task setting was effective. Meanwhile, we measured the WM capacity of participants and excluded the potential confounding of individual’s WM capacities.

We further analyzed the choices on four decks in each WM load group, and results indicated that the number of plays on Deck B in all groups showed the same trend that declined significantly over the course of the task. They all showed preference for Decks C and D, but for disadvantageous Deck A, there was a linear trend in the three groups’ choices, which indicated that WM load had disturbed the participants’ ability to identify Deck A as a disadvantageous deck. This is consistent with Pecchinenda et al.’s (2006) study. They found that for disadvantageous Deck A, WM load had an effect on participants’ choice. But for advantageous Deck C, WM load did not have an effect. That means participants in low WM load group could discriminate Deck A and Deck C, while participants in high WM load group could not discriminate these two decks in their study. In the present study, participants in the no WM load group could discriminate Deck A and Deck C, while participants in the high WM load group could not discriminate these two decks. Since both Deck A and C had high frequency of loss, it was not the case that WM load only affects the participants’ ability to maintain the advantageousness of a deck by keeping the number of wins/losses associated with that deck. Rather the implicit learning of the advantageousness of decks in the IGT relies on WM to maintain multiple information of decks (Pecchinenda et al., 2006). However, it should be cautious to compare our results to previous studies directly, because we used a different version of IGT. Furthermore, even in WM load groups, participants showed avoidance for Deck B in the single choice version, which was consistent with previous studies (Cui et al., 2013, in preparation).

### THE INFLUENCE OF WM LOAD ON IMPLICIT LEARNING EFFECTS AND EXPLICIT COGNITION

In this study, we added a stage in which no single-trial feedback was provided to examine the participants’ implicit learning effects. The results of this stage suggested that net score of this stage was not significantly different among the three groups. Net score of no WM load group and low WM load group was larger than 0, while it was not differently different from 0 in the high WM load group. These results suggested that the implicit learning in the IGT somewhat depended on cognitive resources, and implicit learning might play a role in decision-making.

The analysis on the participants’ explicit cognition of IGT and favorite deck revealed that they all presented explicit knowledge to IGT, which is consistent with previous studies (Maia and McClelland, 2004; Guillaume et al., 2009). Moreover, the explicit cognition in the no WM load group was correlated with IGT performance which was also consistent with Guillaume et al. (2009). We also extended previous studied by showing that the three WM load groups did not show significant difference on explicit cognition of IGT. These results suggested that WM load did not affect participants’ explicit cognition at the end of the task. Nevertheless, since we believe that multiple assessment of explicit cognition might provide clue to subjects and make them aware (Persaud et al., 2007), we just assessed the explicit cognition once at the end of the task. Therefore, we could not know whether there would be difference in explicit cognition in the middle of the task or not. Whether the acquirement of explicit knowledge was also delayed in participants with WM load, like IGT net score, should be studied further.

The participants in the high WM load group were somewhat similar to ventromedial prefrontal lesion patients that they showed explicit cognition to the task. However, they did not follow their knowledge and performed disadvantageously (Bechara et al., 1997). It might be that central executive resources are required for developing somatic markers (Jameson et al., 2004). Participants in the high WM load group whose dorsolateral and ventrolateral prefrontal functions were interfered did not have enough central executive resources, thus they did not develop somatic makers which involved the functions of ventromedial prefrontal cortex to guide them to make advantageous decisions.

Overall, the results of present study indicated that though WM load affect the learning processes, the learning effect of IGT was not entirely dependent on WM since the explicit cognition in the high WM load group reached a similar level as in other two groups. If the WM resource affected the participants’ learning speed, it might be useful to increase the number of trials to provide enough learning experience and to see whether the WM resource is related to the emergence of hunches (Bagneux et al., 2013). In the present study, we had a total of 200 trials and provide enough learning experiences and found out that WM load affected the learning process but not the explicit cognition on IGT.

### IS THE COGNITIVE EXPLANATION ABOUT IGT REASONABLE?

For people’s IGT task performance, Damasio (1994) and Bechara et al. (1997) emphasized the role of body generated emotional signals (somatic states) in guiding the decision-making behavior. Some researchers suggested other explanations (e.g., Fellows and Farah, 2003; Maia and McClelland, 2004). They argued that WM, reversal learning/inhibition, risk preference and other cognitive factors also played a role in IGT performance (see Dunn et al., 2006). In this study, the WM hypothesis was tested and has been partially supported. WM affected the performance and implicit learning but not explicit cognition. Other cognitive explanations such as reversal learning, risk preference were not explored. Our results revealed that other factors might play a role in IGT performance. And our ERP study provided more direct evidence on the role of emotion (Cui et al., 2013).

Previous researches have looked for evidence for these theoretical explanations in special populations (e.g., VMPFC patients Bechara et al., 1998; Fellows and Farah, 2003, 2005b; Sanfey et al., 2003; Shiv et al., 2005; schizophrenia Turnbull et al., 2006; etc.). This study may not respond to these controversies because we only recruited healthy participants. Perhaps as Dunn et al. (2006) suggested, different interpretations did not necessarily mutually exclusive, and different mechanisms might be involved in successful IGT performance. Impairment in any component would affect task performance, and different mechanisms might explain different aspects of tasks or performances in different populations.

There are several limitations in the study. First, study indicated that children with high socioeconomic status performed better on Children’s Gambling Task compared to children with low socioeconomic status (Mata et al., 2013). The socioeconomic status may also influence young adults’ IGT performance. However, the socioeconomic level of the students was not recorded in the present study. Second, Jameson et al. (2004) suggested that the central executive resources were required in the development of somatic markers and IGT performance. Yet we did not record SCRs in this study, as a result we could not make a causal conclusion on the role of WM and somatic makers in IGT performance. Third, the task in the high WM load condition might not be challenging enough, this might cause the marginal effect of WM load on IGT performance.

## CONCLUSION

To summarize, the present study revealed that WM played a partial role in IGT performance. Patients in the high WM load group showed explicit cognition to the task but still decided disadvantageously. The results indicated that it might be consistent with SMH that participants are guided by their emotions when performing the IGT. Nevertheless, this study mainly investigated the effect of WM on the IGT performance. The role of emotion was inferred and indirect. And we suggested further studies should explore the role of emotion in IGT performance more directly.

## Conflict of Interest Statement

The authors declare that the research was conducted in the absence of any commercial or financial relationships that could be construed as a potential conflict of interest.
